# Comparison of Illumina and 454 Deep Sequencing in Participants Failing Raltegravir-Based Antiretroviral Therapy

**DOI:** 10.1371/journal.pone.0090485

**Published:** 2014-03-06

**Authors:** Jonathan Z. Li, Brad Chapman, Patrick Charlebois, Oliver Hofmann, Brian Weiner, Alyssa J. Porter, Reshmi Samuel, Saran Vardhanabhuti, Lu Zheng, Joseph Eron, Babafemi Taiwo, Michael C. Zody, Matthew R. Henn, Daniel R. Kuritzkes, Winston Hide, and the ACTG A5262 Study Team, Cara C. Wilson, Baiba I. Berzins, Edward P. Acosta, Barbara Bastow, Peter S. Kim, Sarah W. Read, Jennifer Janik, Debra S. Meres, Michael M. Lederman, Lori Mong-Kryspin, Karl E. Shaw, Louis G. Zimmerman, Randi Leavitt, Guy De La Rosa, Amy Jennings

**Affiliations:** University of Colorado; Northwestern University; University of Alabama at Birmingham; ACTG Operations Center, Social & Scientific Systems, Inc.; National Institute of Allergy and Infectious Diseases; National Institute of Allergy and Infectious Diseases; Frontier Science & Technology Research Foundation; National Institute of Allergy and Infectious Diseases; Case Western Reserve University; Ohio State University; Henry Ford Hospital CRS; University of Colorado CRS; Merck; Tibotec; ACTG Operations Center; 1 Division of Infectious Diseases, Brigham and Women's Hospital, Harvard Medical School, Boston, Massachusetts, United States of America; 2 Harvard School of Public Health, Boston, Massachusetts, United States of America; 3 Broad Institute of MIT and Harvard, Cambridge, Massachusetts, United States of America; 4 Department of Virology, University of KwaZulu-Natal, National Health Laboratory Service, Durban, South Africa; 5 Division of Infectious Diseases, University of North Carolina, Chapel Hill, North Carolina, United States of America; 6 Division of Infectious Diseases, Northwestern University, Chicago, Illinois, United States of America; Technische Universität Dresden, Germany

## Abstract

**Background:**

The impact of raltegravir-resistant HIV-1 minority variants (MVs) on raltegravir treatment failure is unknown. Illumina sequencing offers greater throughput than 454, but sequence analysis tools for viral sequencing are needed. We evaluated Illumina and 454 for the detection of HIV-1 raltegravir-resistant MVs.

**Methods:**

A5262 was a single-arm study of raltegravir and darunavir/ritonavir in treatment-naïve patients. Pre-treatment plasma was obtained from 5 participants with raltegravir resistance at the time of virologic failure. A control library was created by pooling integrase clones at predefined proportions. Multiplexed sequencing was performed with Illumina and 454 platforms at comparable costs. Illumina sequence analysis was performed with the novel *snp-assess* tool and 454 sequencing was analyzed with *V-Phaser*.

**Results:**

Illumina sequencing resulted in significantly higher sequence coverage and a 0.095% limit of detection. Illumina accurately detected all MVs in the control library at ≥0.5% and 7/10 MVs expected at 0.1%. 454 sequencing failed to detect any MVs at 0.1% with 5 false positive calls. For MVs detected in the patient samples by both 454 and Illumina, the correlation in the detected variant frequencies was high (R^2^ = 0.92, P<0.001). Illumina sequencing detected 2.4-fold greater nucleotide MVs and 2.9-fold greater amino acid MVs compared to 454. The only raltegravir-resistant MV detected was an E138K mutation in one participant by Illumina sequencing, but not by 454.

**Conclusions:**

In participants of A5262 with raltegravir resistance at virologic failure, baseline raltegravir-resistant MVs were rarely detected. At comparable costs to 454 sequencing, Illumina demonstrated greater depth of coverage, increased sensitivity for detecting HIV MVs, and fewer false positive variant calls.

## Introduction

Current commercial genotypic testing for HIV-1 drug resistance are based on PCR amplification and population Sanger sequencing technologies that do not reliably detect the presence of low-frequency resistance mutations present at <15–20% of the viral population [1], [2]. These drug-resistant minority variants (MVs) can significantly increase the risk of antiretroviral treatment (ART) failure, especially for individuals on non-nucleoside reverse transcriptase inhibitor (NNRTI)-based regimens [3], [4]. Advances in next-generation sequencing have revolutionized HIV sequencing and the study of HIV MVs. The most commonly used next-generation sequencing platforms are those developed by 454/Roche and Illumina. The general principle behind both of these technologies lies in the clonal amplification of individual molecules of single-stranded HIV DNA to allow detection of nucleotide synthesis of complementary strands by pyrosequencing (454/Roche) or through fluorescently labeled nucleotides (Illumina). The advantage of next-generation sequencing over traditional Sanger sequencing is the ability to sequence millions of such clonal sequences in parallel, resulting in significant time and cost savings. The 454/Roche system historically has been the most popular platform for HIV applications as it was the first to become commercially available, has a relatively long read length, and is supported by a number of available bioinformatics tools. In contrast, Illumina sequencing offers significantly greater throughput than 454 and has become the most popular deep sequencing platform across all applications [5]. However, the lack of well-validated viral sequence analysis tools for the Illumina platform remains a hurdle to the wide-spread adoption of Illumina for HIV applications.

Raltegravir is an integrase strand-transfer inhibitor (INSTI) and one of the preferred first-line antiretroviral medications for treatment-naïve individuals [6]. Resistance to raltegravir shares a number of characteristics with NNRTI resistance that suggests a role for raltegravir-resistant MVs in increasing the risk of virologic failure. For example, single amino acid changes can confer significant resistance to raltegravir, suggesting a low barrier to resistance. As with NNRTIs, clinical failure of raltegravir is commonly accompanied by genotypic evidence of drug resistance [7]. In addition, virologic failure and emergence of raltegravir resistance have been reported in a patient with pre-existing raltegravir-resistant MVs [8]. Despite the detection of primary or secondary raltegravir-resistant MVs in a subset of patients prior to raltegravir exposure, evidence is still lacking that these MVs increase the risk of raltegravir treatment failure [9], [10], [11].

We compared the performance of Illumina and 454 in the detection of HIV-1 MVs in a control library and from pre-treatment samples of patients in whom raltegravir-resistant mutants were detected at the time of virologic failure. The two main aims of this study are to compare Illumina and 454 sequencing for HIV MV detection and to assess whether raltegravir-resistant MVs may have contributed to the treatment failure of patients on a raltegravir-based ART regimen.

## Materials and Methods

### Patients and Study Design

ACTG A5262 (NCT00830804) was a single-arm study of raltegravir and darunavir/ritonavir in treatment-naïve patients [12]. Patients with more than one darunavir resistance-associated mutation or with known major integrase resistance-associated mutations (N155H, Q148H/R/K, Y143C/R, and G140S) were excluded from the study. Of the 112 participants who initiated treatment, 5 participants had detectable raltegravir resistance mutations by population sequencing at the time of virologic failure. Pre-treatment plasma were obtained from these 5 participants for evaluation of baseline raltegravir-resistant MVs. All samples had previously measured viral load >100,000 copies/mL. All participants provided written informed consent and this study was approved by the Partners institutional review board.

### PCR Amplification and Control Library Construction

Stored plasma samples (3 ml) from the five A5262 participants were ultracentrifuged at 28,000×g for 1 hour to pellet virus prior to RNA extraction (QIAamp viral RNA minikit). Synthesis of cDNA was performed using an integrase-specific primer and the Superscript III reverse transcriptase. A 401 base pair region of the HIV-1 integrase gene (HXB2 nucleotides 4374–4774) was PCR amplified using a conserved, nested primer set. Each PCR amplification step was performed in quadruplicate using PfuUltra II DNA polymerase. The number of full-length template copy numbers was estimated after the cDNA synthesis step by using SYBR green real-time PCR and primers targeting the 5′ end of the region of interest.

The accuracy of the deep sequencing platforms were evaluated with a control library of clonal HIV sequences mixed at known concentrations. PCR amplicons from each patient and from the HXB2 reference strain were inserted into a pCR4-TOPO plasmid vector (Invitrogen). The HXB2 reference strain and one HIV-1 integrase clone from each patient were selected for PCR amplification using the high fidelity PfuUltra II DNA polymerase (Agilent) and T3/T7 primers. The PCR amplicons were gel purified and quantified by Nanodrop spectrophotometry. A control library was created by mixing the clones at concentrations of 60%, 33.4%, 5%, 1%, 0.5%, and 0.1%.

### Illumina Deep Sequencing and Sequence Analysis

Illumina library construction and sequencing of the control library and 5 patient samples were performed at the Partners Healthcare Center for Personalized Genetic Medicine using the Illumina HiSeq 2000 platform. The library construction process was optimized for short amplicon size using an extended DNA shearing time. The Illumina sequence analysis pipeline (*snp-assess*) was created in the Center for Health Bioinformatics at the Harvard School of Public Health and is publicly available (https://github.com/hbc/projects/tree/master/snp-assess). Reads containing undefined nucleotides (’N's) were filtered out. To avoid aligning identical reads multiple times remaining FASTQ reads with identical sequence information were collapsed into unique representations, using the best base quality information from all identical reads as base quality for the unique read. Unique reads were aligned to the consensus reference sequence using NovoAlign (Novocraft Technologies) with default parameters. Aligned reads were re-aligned using the GATK framework [13] to minimize inconsistent and incorrect alignments due to indels. To differentiate low-frequency variations from likely sequencing errors we described unique reads at each position with their a) quality score (the Phred score of sequencing quality at a base, assigned by the sequencer), b) mapping score (the alignment score of a read, assigned by the Novoalign aligner) and c) k-mer frequency (the frequency of the 13 bp region surrounding a position). Based on the outcome of a TopCoder crowdsourcing competition (http://community.topcoder.com/longcontest/?module=ViewProblemStatement&compid=24758&rd=15080) [14], we implemented a random-forest classifier using a combinations of these three metrics to filter out likely false positive variants before calculating variant frequency based on the remaining unique reads and their associated original read counts. The MV limit of detection was calculated as the threshold that removed 99% of false positive MVs in the control library. These false positive MVs were identified at positions where no MVs were expected in the control library.

A down-sampling analysis was performed using the control library dataset to determine the assay characteristics at lower coverage rates by randomly removing unique reads to generate different coverage depths prior to assessing false positive and negative variant calls. A total of ten boot-strapping iterations were performed at each coverage depth to determine the standard deviations.

The Illumina variant calling algorithm (*snp-assess*), including classifiers and training data is available at https://github.com/hbc/projects/tree/master/snp-assess. A set of scripts providing an automated pipeline for identifying mutations in viral populations using Illumina deep sequencing is available at https://github.com/hbc/projects/tree/master/jl_hiv along with installation scripts and dependencies. Illumina sequencing data has been deposited in the European Nucleotide Archive under study accession number PRJEB5053 (http://www.ebi.ac.uk/ena/data/view/PRJEB5053).

### 454/Roche Deep Sequencing and Sequence Analysis

454 library construction was performed at the Broad Institute of MIT and Harvard (Cambridge, MA) using the same PCR amplicon starting product as the Illumina library construction. Multiplexed sequencing was performed at the Broad Institute using the GS-FLX platform (approximately the same cost as the Illumina sequencing run: ∼$1200/sample for 454 and ∼$800/sample for Illumina) and at Roche (Branford, CT) on a GS Junior platform. 454 sequence analysis was performed with *V-Phaser*, software designed for rare variant detection in mixed viral populations [15]. For the cross-platform comparisons, *V-Phaser* variant calls below the Illumina limit of detection were excluded from the analysis.

In brief, reads for each sample were aligned to a portion the HXB2 reference genome (K03455.1) from position 3596 to 3996 using Mosaik (version 1.0.1388, github.com/wanpinglee/MOSAIK). Alignments outside the amplified region were ignored. Reads were cleaned of carry-forward and incomplete extension (CAFIE) and homopolymer/frameshift errors using RC454 [16]. After cleaning, reads were realigned with Mosaik. The alignments were passed to *V-Phaser* [15] for variant calling. Briefly, *V-Phaser* uses an autocalibration model to recalibrate quality scores for individual bases. After recalibration it then uses a combined pileup and two-site phasing model to identify positions or pairwise combinations of positions that have more minor alleles than would be expected at random accounting for the error probabilities predicted by the recalibrated base quality. Variant frequencies were then estimated based on the proportional observations of all valid alleles at each position, ignoring any reads that presented an allele at a given position that was not listed as a valid allele in the initial *V-Phaser* call set. 454 data is available at: http://trace.ncbi.nlm.nih.gov/Traces/study/?acc=ERP004411.

### Statistical Analysis

Linear regression slopes, 95% confidence intervals, and goodness of fit (R^2^) were calculated and plotted to compare measured and expected MV frequencies for the control library and to compare the frequency of MVs detected in the patient samples across platforms. The false positive rate was calculated by dividing the number of false positive MV calls by the number of nucleotides in the amplicon excluding the primer sequences (354 nucleotides). Bland-Altman plots were used to further assess the level of agreement between platforms by plotting the percent difference in MV frequencies between Illumina and 454 against the average of the two measurements.

## Results

### Illumina and 454 Coverage and Test Characteristics

All 5 patient samples were confirmed to have >100,000 HIV-1 cDNA template copies (range 136,000 to 444,000 copies/mL, Table S1). The median Illumina coverage of each nucleotide position was 2.8 million [IQR 1.8–6.2 million] for the five A5262 patient samples and 2.2 million [IQR 1.8–4.2 million] for the control library. The median 454 coverage for each nucleotide position was more than 1,000-fold lower: 1349 [IQR 1093–1692] for the five A5262 patient samples and 2349 [IQR 2348–2350] for the control library.

The Illumina limit of detection was calculated to be 0.095%, removing ≥99% of potential false positives. The *VPhaser* algorithm uses position-specific factors to make variant calls and does not calculate a comparable overall limit of detection for the 454 data. Illumina sequencing of the control library accurately detected all nucleotide MVs present at ≥0.5% and 7 of 10 MVs present at 0.1% (Table 1). One false positive nucleotide MV was detected at 0.2% frequency in the control library (0.3% false positive rate amongst all 354 nucleotide positions in the amplicon). By contrast, 454 sequencing detected only 8 of 10 MVs present at 1% and 0 of 10 MVs expected to be present at 0.1%. 454 sequencing also had a significantly higher false positive rate with 5 false positive MVs detected (1.4% false positive rate) at frequencies ranging from 0.09% to 0.6%. Similar results were obtained when analyzing the data at the amino acid level (Table 2). Illumina detected all expected amino acid MVs with the exception of 1 of 4 MVs expected at 0.1%. On the other hand, 454 failed to detect 1 of 2 amino acid MVs present at 1% and all 4 of the MVs expected at 0.1%. The numbers of false positive MVs calls remained unchanged from the nucleotide analysis.

**Table 1:** Performance of Illumina and 454 sequencing for the detection of nucleotide minority variants within the control library.

*Expected Variant %*	*N*	*Median Variant % by Illumina*	*Missed by Illumina (FN)*	*Median Variant % by 454*	*Missed by 454 (FN)*
**100%**	299	100%	0	100%	0
**40**	2	55.2	0	32.0	0
**39.9**	1	32.4	0	31.7	0
**39.5**	1	44.4	0	31.1	0
**38.9**	1	44.5	0	31.0	0
**38.4**	2	47.2	0	30.3	0
**34.4**	2	37.9	0	26.2	0
**34**	1	30.0	0	25.6	0
**33.5**	1	29.1	0	25.1	0
**33.4**	8	30.2	0	25.2	0
**6.6**	1	8.2	0	6.9	0
**6.1**	1	7.8	0	6.4	0
**5.1**	1	6.1	0	5.3	0
**5.0**	8	6.1	0	5.3	0
**1.0**	10	1.4	0	1.2	2
**0.6**	1	0.6	0	0.5	0
**0.5**	8	0.5	0	0.5	0
**0.1**	10	0.2	3	n/a	10

The control library was created by mixing 6 HIV-1 integrase clones at concentrations of 60%, 33.4%, 5%, 1%, 0.5%, and 0.1%. Expected variant percentages include positions where a MV is present on more than one clone. Median variant % reflects only the minority variants detected by each platform and does not include the undetected variants. N represents the number of nucleotide positions in the control library where the variant frequency is expected. Illumina detected 1 false positive minority variant present at 0.2% of the viral population (0.3% false positive rate) and 454 detected 5 false positive minority variants ranging from 0.09% to 0.6% (1.4% false positive rate). FN, false negative.

**Table 2:** Performance of Illumina and 454 sequencing for the detection of amino acid minority variants within the control library.

*Expected Variant %*	*N*	*Median Variant % by Illumina*	*Missed by Illumina (FN)*	*Median Variant % by 454*	*Missed by 454 (FN)*
**100%**	114	100%	0	100%	0
**39.9**	1	32.5	0	31.7	0
**33.9**	1	38.4	0	25.6	0
**33.4**	1	27.2	0	25.2	0
**5.1**	1	6.6	0	5.3	0
**5.0**	6	6.1	0	5.3	0
**1.0**	2	1.4	0	1.2	1
**0.5**	1	0.3	0	0.6	0
**0.1**	4	0.2	1	n/a	4

Expected variant percentages include positions where a MV is present on more than one clone. Median variant % reflects only the minority variants detected by each platform and does not include the undetected variants. N represents the number of nucleotide positions in the control library where the variant frequency is expected. Illumina detected 1 false positive minority variant present at 0.7% of the viral population and 454 detected 5 false positive minority variants ranging from 0.09% to 0.6%. FN, false negative.

**Figure 1:**
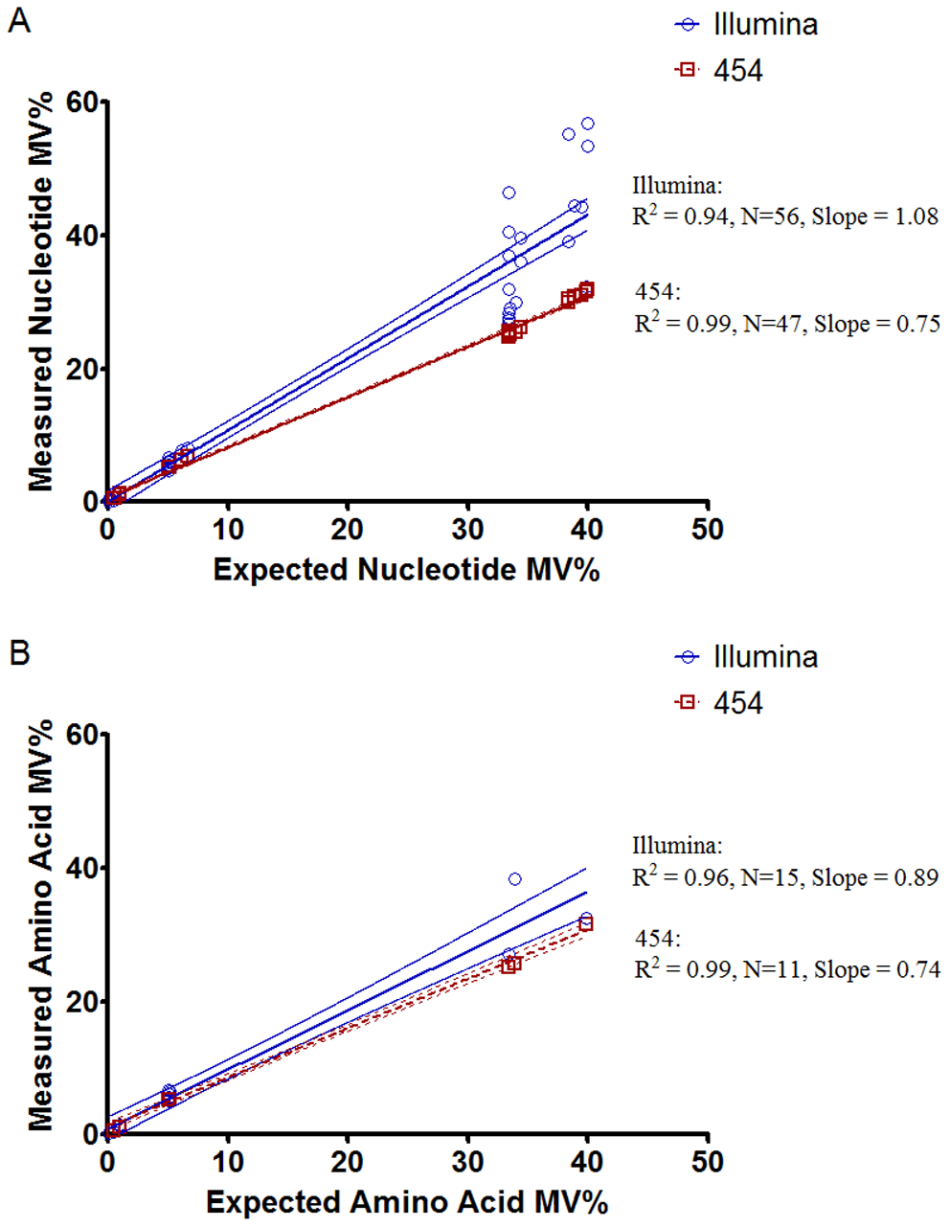
Measured versus expected minority variant percentages detected in the control library by Illumina and 454 sequencing. (A) Nucleotide percentages are plotted with linear regression line and 95% confidence intervals. (B) Amino acid percentages are plotted with 95% confidence intervals. MV, minority variant.

In addition, the frequencies of the MVs detected by Illumina were significantly closer to the expected frequencies when compared to the frequencies detected by 454 sequencing (nucleotide: Illumina slope  = 1.08 [95% CI 1.03–1.11] vs. 454 slope 0.75 [95% CI 0.74–0.76]; amino acid: Illumina slope  = 0.89 [95% CI 0.78–1.0] vs. 454 slope 0.74 [95% CI 0.71–0.77]; Figure 1).

### A5262 Patient Samples

**Figure 2:**
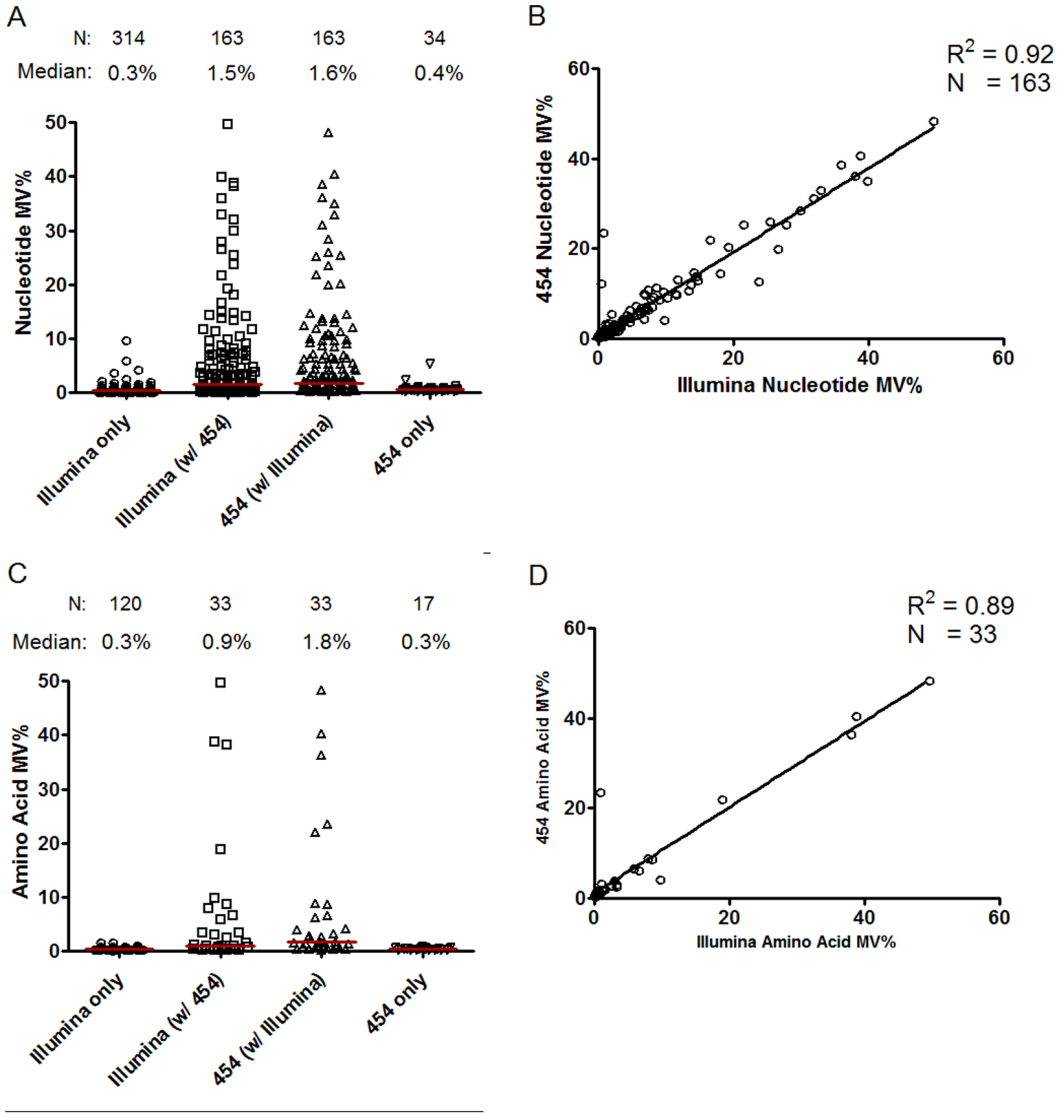
Minority variants detected by Illumina and/or 454 sequencing in the 5 patient samples combined. (A) Nucleotide minority variants categorized by platform (Illumina vs. 454) and whether the minority variants were detected by both Illumina and 454 or by one platform only. The “Illumina (w/454)” category refers to the Illumina minority variant calls that are also detected by 454 and the “454 (w/Illumina)” category refers to the 454 calls that are also detected by Illumina. (B) Pearson correlation of the nucleotide minority variants detected by both Illumina and 454. (C) Amino acid minority variants categorized by platform and whether the variants were detected by both Illumina and 454 or by one platform only. (D) Pearson correlation of the amino acid minority variants detected by both Illumina and 454. MV, minority variant.

Illumina sequencing detected 2.4-fold more nucleotide MVs and 2.9-fold more amino acid MVs compared to 454 sequencing (Figure 2a and 2c, Illumina vs. 454: 477 vs. 197 for nucleotide MVs and 153 vs. 53 amino acid MVs, respectively). The MVs detected by both Illumina and 454 were present at higher frequencies than those detected by only a single platform. The frequencies of MVs detected by both 454 and Illumina in the 5 patient samples were highly correlated (nucleotide: R^2^ = 0.92, P<0.001, N = 163; amino acid: R^2^ = 0.89, P<0.001; Figure 2b and 2d, respectively). At the lower MV frequencies, the Bland-Altman plot showed that 454 tended to report higher frequencies compared to Illumina, especially for the amino acid analysis (Figure S1). We also manually inspected nine nucleotide positions where MVs were detected for at least one patient at relatively high frequency (>1%) by 454, but not by Illumina sequencing. All of these sites were either adjacent to homopolymers or had evidence of strand bias that were indicative of artifact.

The only raltegravir-resistant MV detected was an E138K mutation detected at a frequency of 0.15% in one participant by Illumina sequencing, but not by 454. This mutation was not detected by standard genotyping at the time of virologic failure.

### Down-sampling Illumina Sequence Coverage and Increasing 454 Coverage

**Figure 3:**
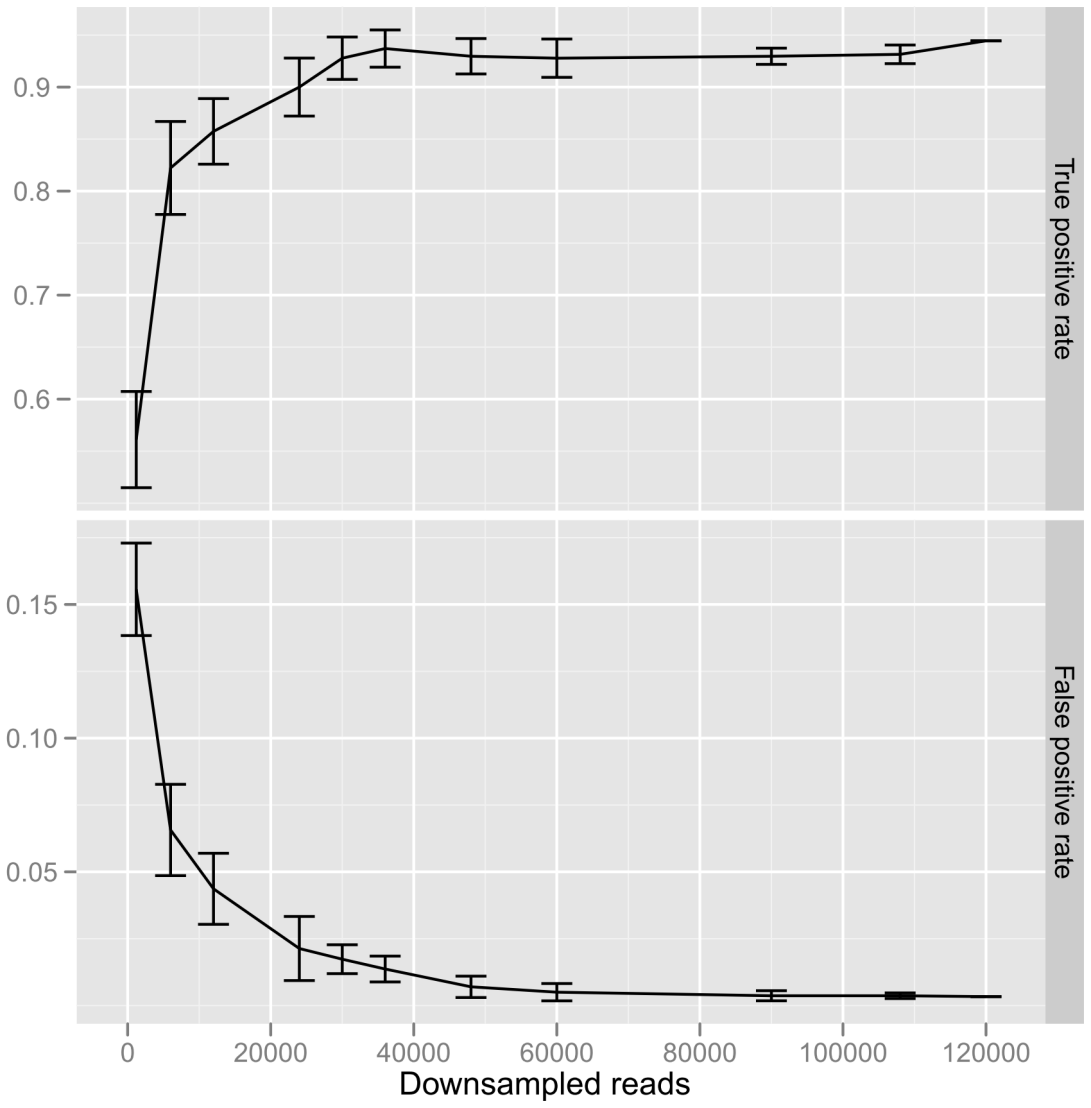
Effect of down-sampling Illumina coverage on true positive and false positive rates. Plots are of the median true positive and false positive minority variant detection rates of the control library with the standard deviation shown as error bars from 10 iterations of random sampling from all reads to generate each coverage depth.

The effect of down-sampling the Illumina coverage level was performed by 10 iterations of random sampling from all control library reads to generate the varying coverage depth. This analysis showed that the true positive and false positive rates remained relatively constant down to 60,000× coverage, which implies that <10% of the observed nucleotide coverage was needed to produce similar Illumina assay characteristics (Figure 3).

For one of the A5262 patient libraries, we repeated the 454 sequencing at a much higher read coverage. The median coverage of each nucleotide position was increased from 2282 on the first run to 6384 on the repeat. We found a 2-fold increase the number of nucleotide MVs (from 49 to 98). The number of MVs detected by both Illumina and 454 increased from 38 to 57, resulting in far fewer nucleotide MVs detected by Illumina alone (64 MVs in run 1 vs. 45 MVs in run 2). When a similarly increased 454 coverage was used to detect MVs in the control library, the number of 0.1% MVs detected increased from 0 to 6 out of the 10 expected positions. However, the number of false positive MVs reported by *V-Phaser* also increased substantially (1.4% to 8.5% false positive rate).

## Discussion

This study had two main goals: to compare the Illumina and 454/Roche platforms for HIV deep sequencing and to evaluate the presence of raltegravir-resistant HIV-1 MVs in participants of ACTG A5262 in whom raltegravir resistance mutations were detected by standard population sequencing at the time of virologic failure. Using a novel Illumina sequence analysis pipeline (*snp-assess*), we found that at comparable cost, Illumina provided >1,000× greater nucleotide coverage compared to 454 as analyzed by the *V-Phaser* software. In addition, Illumina sequencing provided increased sensitivity for detecting HIV MVs and reported fewer false positive variants than did 454 sequencing. A down-sampling analysis showed that similar rates of Illumina false positive and false negative MV detection could be achieved with <10% of the nucleotide coverage rates used in the current study. This finding suggests that significantly more samples can be multiplexed on an Illumina flow cell without sacrificing accuracy, dramatically enhancing the cost-savings available with Illumina sequencing compared to 454. Increasing the depth of coverage by 454 improved MV detection in the patient samples, but at significantly higher cost.

One of the hurdles to the wide-spread adoption of Illumina sequencing has been the need to validate Illumina viral sequencing and sequencing analysis pipelines against those available for the more established 454 platform. For that reason, we decided to compare Illumina sequencing results against 454 sequencing and the *V-Phaser* sequence analysis algorithm. *V-Phaser* is a variant calling package that uses both phase information and base quality to optimize the accuracy of variant calls for highly diverse viral genomes. It achieves >97% sensitivity and specificity and compares favorably to other commonly-used 454 viral variant callers [15]. The results of this study support the findings of the few previous reported comparisons of Illumina and 454 for HIV sequencing. One study used a clonal control library to compare the ability of Illumina and 454 sequencing to estimate viral diversity estimation and perform haplotype reconstruction. That study found higher accuracy and throughput with Illumina, but advantages with 454 in haplotype reconstruction [17]. A second study compared four different deep sequencing platforms to predict HIV-1 coreceptor tropism [18]. Compared to 454, Illumina had similar rates of substitution errors, but 20-fold lower deletion errors. A few reports have compared Illumina and 454 for the sequencing of bacterial and non-HIV viral genomes [19],[20]. In those studies, the two platforms showed excellent concordance in detected variant frequencies, but Illumina demonstrated fewer insertions/deletions and significant cost savings.

The direct comparison of next generation sequencing platforms can be challenging given differences in sample preparation and analysis. Unlike some of the previously described studies, we controlled for PCR-related errors generated in the process of amplifying the control library and patient HIV sequences by using one set of conserved primers and PCR reactions. The resulting amplicons were split into two samples, one used for Illumina and one for 454 library generation and sequencing. We then adapted the Illumina library preparation process for processing short amplicons with excellent resulting sequence coverage. The direct comparison of next-generation platforms is also complicated by dramatic intrinsic differences in throughput and read coverage. In this study, Illumina sequencing produced >1,000× the sequence coverage of 454 despite similar commercial sequencing costs. We believe that comparing the sequencing results produced at similar “real world” cost would best reflect the choices available to the average user. In addition, we performed a down-sampling analysis showing that Illumina assay characteristics could be replicated using <10% of the observed coverage rates. This approach should allow for increased multiplexing on the Illumina platform without sacrificing accuracy and significantly decreasing costs.

There are a number of shared characteristics of raltegravir and NNRTIs that suggests a potential role for drug-resistant MVs in elevating the risk of virologic failure (e.g., low genetic barrier of resistance, resistance frequently detected at the time of virologic failure). However, using two highly sensitive methods of deep sequencing, we found that raltegravir-resistant MVs were rarely detected prior to initiating antiretroviral therapy, even in patients in whom raltegravir resistance mutations were detected at the time of treatment failure. Whereas one case report showed the emergence of raltegravir resistance in a patient with baseline MVs [8], a number of other studies have failed to detect a significant association between the presence of raltegravir-resistant MVs and risk of virologic failure [9], [10], [11], [21], [22]. However, those studies were limited by the number of resistance sites that could be evaluated using mutation-specific assays (e.g., allele-specific PCR) or by the cost and relatively high limit of variant detection associated with 454 deep sequencing. With the validation of Illumina for HIV sequencing, the comprehensive evaluation of HIV drug-resistant MVs in integrase and other HIV-1 genes should become increasingly cost-effective and feasible for significantly larger studies.

This study has a few notable limitations. Assay characteristics for next-generation sequencing platforms are dependent on the sequence analysis pipeline. We chose to compare a novel Illumina pipeline with an existing 454 analysis system (*V-Phaser*) for several reasons. First, the Illumina sequence analysis pipeline has not been optimized to correct for some errors commonly produced in 454 sequencing (e.g., homopolymers and carry-forward/incomplete extension errors) while the *V-Phaser* system is not yet able to process Illumina sequencing data due to memory and run-time constraints in its current implementation. Another challenge in comparing the results of the two pipelines is that *V-Phaser* does not report an overall limit of MV detection as it relies on phasing between observed variants that is position-specific [15]. For a more direct comparison of the platforms, we excluded MVs called by *V-Phaser* that were below the Illumina limit of detection. While we found that Illumina has advantages in variant detection, 454 generates significantly longer reads and has advantages in haplotype reconstruction and linkage analysis that are not part of the current analysis. While the majority of the 454 sequencing was performed using the GS FLX system, a subset of the data was generated with the GS Junior system (e.g., to evaluate the impact of higher 454 read coverage). The sequencing chemistry is identical between the two 454/Roche platforms and the performance of both instruments has been shown to be nearly identical as well. Finally, Illumina sequencing of the control library demonstrated excellent sensitivity of detection, low rate of false positive variant calls, and high concordance with expected MV frequencies despite not using additional methods for controlling for PCR-induced errors such as Primer ID [23]. The use of Primer ID may have further improved the Illumina error rate, but may be of greatest benefit in controlling for PCR-induced recombination events during variant linkage analysis.

Using a novel and now publicly-available sequence analysis software, we found that Illumina sequencing demonstrates greater depth of coverage, increased sensitivity for detecting HIV MVs, and fewer false positive variant calls compared to 454 sequencing performed at similar costs. In participants of A5262 with raltegravir resistance at virologic failure, Illumina and 454 sequencing showed that baseline raltegravir-resistant MVs were rarely detected. Larger studies are needed to evaluate more fully the role, if any, of integrase inhibitor-resistant MVs in determining treatment outcome.

## Supporting Information

Figure S1 **Bland-Altman analysis of minority variant frequencies detected by Illumina and 454 deep sequencing in the 5 patient samples.** The *y*-axis shows the percent difference in minority variant frequency measurements between Illumina and 454 results and the *x*-axis shows the average of the two measurements for (A) nucleotide and (B) amino acid minority variant analysis. Only minority variants identified by both platforms were included in this analysis. Dotted lines represent 95% limits of agreement.
(TIF)

Table S1 **Expected plasma HIV-1 RNA viral loads and measured full-length cDNA template copies used for deep sequencing library preparation.** The viral loads were previously measured as part of the A5262 study. The number of full-length template copy numbers used for deep sequencing was measured after the cDNA synthesis step.
(DOCX)

Table S2 Sequences of the clones used for the control library.
(DOCX)
