# Supplementary figures and images for: Comparison of Illumina and 454 Deep Sequencing in Participants Failing Raltegravir-Based Antiretroviral Therapy
Source: PLoS One. 2014 Mar 6;9(3):e90485. doi: 10.1371/journal.pone.0090485 (PMC3946168; doi:10.1371/journal.pone.0090485)

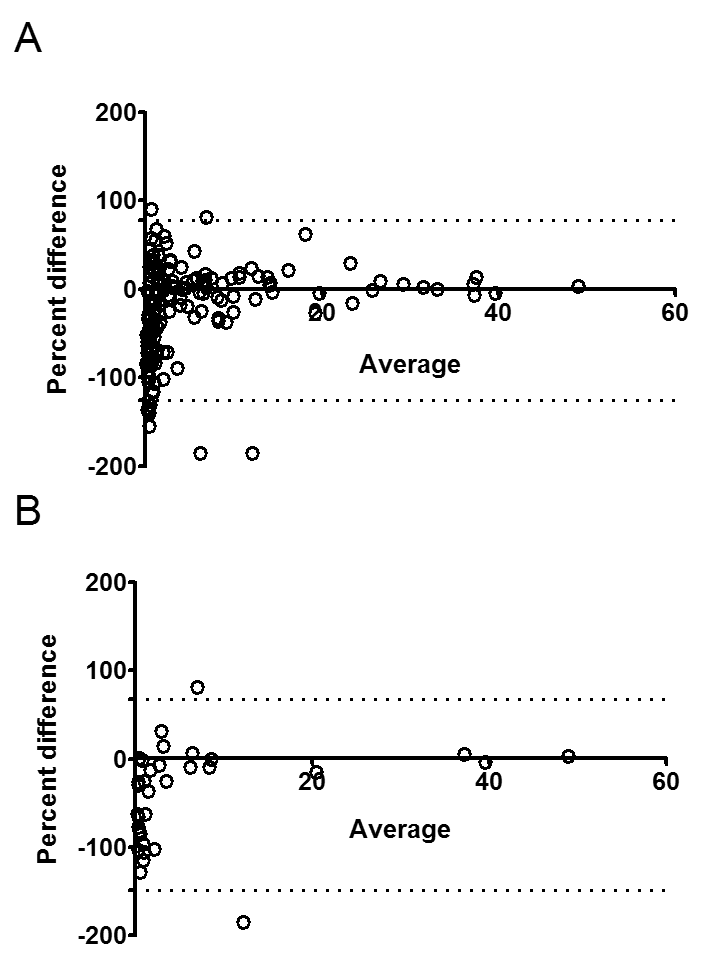

Supplement: Figure S1 — Bland-Altman analysis of minority variant frequencies detected by Illumina and 454 deep sequencing in the 5 patient samples. The y-axis shows the percent difference in minority variant frequency measurements between Illumina and 454 results and the x-axis shows the average of the two measurements for (A) nucleotide and (B) amino acid minority variant analysis. Only minority variants identified by both platforms were included in this analysis. Dotted lines represent 95% limits of agreement. (TIF) [file pone.0090485.s001.tif]
