# Supplementary material for: Comparison of Illumina and 454 Deep Sequencing in Participants Failing Raltegravir-Based Antiretroviral Therapy
Source: PLoS One. 2014 Mar 6;9(3):e90485. doi: 10.1371/journal.pone.0090485 (PMC3946168; doi:10.1371/journal.pone.0090485)
Supplement: Table S1 — Expected plasma HIV-1 RNA viral loads and measured full-length cDNA template copies used for deep sequencing library preparation. The viral loads were previously measured as part of the A5262 study. The number of full-length template copy numbers used for deep sequencing was measured after the cDNA synthesis step. (DOCX) [file pone.0090485.s002.docx]

**Table S1**. Expected plasma HIV-1 RNA viral loads and measured full-length cDNA template copies used for deep sequencing library preparation. The viral loads were previously measured as part of the A5262 study. The number of full-length template copy numbers used for deep sequencing was measured after the cDNA synthesis step.

| ***Subject*** | ***HIV-1 RNA copies/mL*** | ***cDNA template copies/mL*** | ***% Yield*** |
| --- | --- | --- | --- |
| 1 | 245259 | 194568 | 79% |
| 2 | 139308 | 138136 | 99% |
| 3 | 372474 | 204280 | 55% |
| 4 | 1023293 | 136341 | 13% |
| 5 | 159547 | 444019 | 278% |
