# Supplementary material for: Comparison of Illumina and 454 Deep Sequencing in Participants Failing Raltegravir-Based Antiretroviral Therapy
Source: PLoS One. 2014 Mar 6;9(3):e90485. doi: 10.1371/journal.pone.0090485 (PMC3946168; doi:10.1371/journal.pone.0090485)
Supplement: Table S2 — Sequences of the clones used for the control library. (DOCX) [file pone.0090485.s003.docx]

**Table S2**. Sequences of the clones used for the control library.

| ***% of Library*** | ***Sequence*** |
| --- | --- |
| 60% | GCCATGCATGGACAAGTAGACTGTAGTCCAGGAATATGGCAACTAGATTGTACACATTTAGAAGGAAAAGTTATCCTGGTAGCAGTTCATGTAGCCAGTGGATATATAGAAGCAGAAGTTATTCCAGCAGAAACAGGGCAGGAAACAGCATATTTTCTTTTAAAATTAGCAGGAAGATGGCCAGTAAAAACAATACATACAGACAATGGCAGCAATTTCACCAGTGCTACGGTTAAGGCCGCCTGTTGGTGGGCGGGAATCAAGCAGGAATTTGGAATTCCCTACAATCCCCAAAGTCAAGGAGTAGTAGAATCTATGAATAAAGAATTAAAGAAAATTATAGGACAGGTAAGAGATCAGGCTGAACATCTTAAGACAGCAGTACAAATGGCAGTATTCAT |
| 33.4% | GCCATGCATGGACAAGTAGACTGTAGTCCAGGAATATGGCAGCTAGATTGTACACATTTAGAAGGAAAAATTATCCTGGTAGCAGTGCATGTAGCCAGTGGATATATAGAAGCAGAAGTTATCCCAGCAGAGACAGGGCAAGAAACAGCATACTTTCTCCTAAAACTAGCAGGAAGATGGCCAGTAAAAACAATACATACAGACAATGGCAGCAATTTCACCAGTACTACAGTTAAGGCCGCCTGTTGGTGGGCGGGGATCAAGCAGGAATTTGGTATTCCCTACAATCCTCAAAGTCAAGGAGTAATAGAATCTATGAATAAAGAATTAAAGAAGATTATAGGGCAAGTAAGAGATCAGGCTGAACATCTTAAGACAGCAGTACAAATGGCAGTATTCAT |
| 5% | GCCATGCATGGACAAGTAGACTGTAGTCCAAGAATATGGCAACTAGATTGTACACATTTAGAAGGAAAAATTATCCTGGTAGCAGTTCATGTAGCCAGTGGATATATAGAAGCAGAAGTTATTCCAGCAGAAACAGGGCAGGAAACAGCATACTTTCTCTTAAAACTAGCAGGAAGATGGCCAGTAAAAACAATACATACAGACAATGGCACCAATTTCACCAGTAATGCTGTTAAGGCCGCCTGTTGGTGGGCAGGGATCAAGCAAGAATTTGGCATTCCCTACAATCCCCAAAGTCAAGGAGTAGGAGAATCTATGAATAATGAATTAAAGAAAATTATAGGACAAGTAAGAGATCAGGCCGAACATCTTAAGACAGCAGTACAAATGGCAGTATTCAT |
| 1% | GCCATGCATGGACAAGTAGACTGTAGTCCAGGAATATGGCAACTAGATTGTACACACTTAGAAGGGAAAATTATCTTGGTAGCAGTTCATGTAGCCAGTGGATATATAGAAGCAGAAGTTATTCCAGCAGAAACAGGGCAAGAAACAGCATACTTTATCTTAAAATTAGCAGGAAGATGGCCAGTAAAAACAATACATACAGACAATGGCAGCAATTTCACCAGTGCTACAGTGAAGGCCGCCTGTTGGTGGGCAGGGATCAAGCAGGAATTTGGCATTCCCTACAATCCCCAAAGTCAAGGAGTAGTAGAATCTATGAATAAAGAATTAAAACAAATTATAGGACAGGTGAGAGATCAAGCTGAACATCTTAAAACAGCAGTACAAATGGCAGTATTCAT |
| 0.5% | GCCATGCATGGACAAGTAGACTGTAGTCCAGGAATATGGCAATTAGATTGTACACATTTAGAGGGAAAAATCATCCTGGTAGCAGTTCATGTAGCCAGTGGATATATAGAAGCAGAAGTTATTCCAGCAGAGACAGGACAGGAAACAGCATACTTTCTCTTAAAATTAGCAGGAAGATGGCCAGTAAAAATAATACATACAGATAATGGCAGCAATTTCACCAGTACTACGGTTAAGGCCGCCTGTTGGTGGGCGGGAATCAAGCAGGAATTTGGCATCCCCTACAATCCCCAAAGTCAAGGTGTAGTAGAATCTATGAATAAAGAATTAAAGAAAATTATAGGACAGGTAAGAGATCAGGCTGAACATCTCAAGACAGCAGTACAAATGGCAGTATTCAT |
| 0.1% | GCCATGCATGGACAAGTAGACTGTAGTCCAGGAATATGGCAACTAGATTGTACCCATTTAGAAGGAAAAGTTATCCTAGTAGCAGTACATGTAGCCAGTGGATATATAGAAGCAGAAGTTATCCCAGCAGAGACAGGACAGGAAACAGCATACTTTCTCTTAAAATTGGCAGGAAGATGGCCAGTAAGAACAATACATACAGACAATGGCAGCAACTTCACCAGTGGTGCGGTTAAAGCCGCCTGTTGGTGGGCAGGGATCAAGCAGGAATTTGGCATTCCCTACAATCCCCAAAGTCAAGGAGTAGTAGAATCTATGAATAAAGAATTAAAGAACATTATAGGACAGGTAAGAGAACAGGCTGAACATCTTAAGACAGCAGTACAAATGGCAGTATTCAT |
